# How to enhance and assess reflection in specialist training: a mixed method validation study of a new tool for global assessment of reflection ability

**DOI:** 10.1186/s12909-020-02256-5

**Published:** 2020-10-08

**Authors:** Gunver Lillevang, Helle Ibsen, Søren Hast Prins, Niels Kristian Kjaer

**Affiliations:** 1 Department of Clinical Medicine, University of Copenhagen, Blegdamsvej 3B, 2200 Copenhagen N, Denmark; 2 The Research Unit of General Practice, Department of Public Health, Faculty of Health Sciences, University of Southern Denmark, Campusvej 55, 5230 Odense M, Denmark; 3 Centre for Health Sciences Education, Health, Aarhus University, Palle Juul-Jensens Boulevard 82, 8200 Aarhus N, Denmark

**Keywords:** Educational assessment, Reflection, Medical postgraduate education, General practice

## Abstract

**Background:**

In Danish GP training we had the ambition to enhance and assess global reflection ability, but since we found no appropriate validated method in the literature, we decided to develop a new assessment tool. This tool is based on individual trainee developed mind maps and structured trainer-trainee discussions related to specific complex competencies. We named the tool Global Assessment of Reflection ability (GAR) and conducted a mixed method validation study. Our goal was to investigate whether it is possible to enhance and assess reflection ability using the tool.

**Methods:**

In order to investigate acceptability, feasibility, face validity, and construct validity of the tool we conducted a mixed method validation study that combined 1) qualitative data obtained from 750 GP trainers participating in train-the-trainer courses, 2) a questionnaire survey sent to 349 GP trainers and 214 GP trainees and 3) a thorough analysis of eight trainer-trainee discussions.

**Results:**

Our study showed an immediate high acceptance of the GAR tool. Both trainers and trainees found the tool feasible, useful, and relevant with acceptable face validity. Rating of eight audio recordings showed that the tool can demonstrate reflection during assessment of complex competencies.

**Conclusions:**

We have developed an assessment tool (GAR) to enhance and assess reflection. GAR was found to be acceptable, feasible, relevant and with good face- and construct validity. GAR seems to be able to enhance the trainees’ ability to reflect and provide a good basis for assessment in relation to complex competencies.

## Background

Clinical practice is never simple and straight forward. Doctors are practicing complex competencies in a clinical world embedded with uncertainty and where textbook knowledge only provides some of the answers [1, 2]. Clinical decision making therefore requires that doctors can combine experience-based knowledge with evidence-based knowledge and that doctors can constructively process all kinds of formal and informal feedback [3]. Furthermore in a clinical setting inherited with complexity, uncertainty, and time constraint, the doctors often have to shift between analytical and non-analytical decision making [3]. The non-analytical clinical decision making is efficient but also error prone due to decision biases [4–6]. The only way to compensate these decision biases is through deliberate reflection [5, 7]. Hence, doctors’ ability to reflect is crucial for clinical practice and should therefore be addressed in medical education. Yet the concept “reflection” is not unequivocally defined in medical literature, and when we start to discuss how to teach reflection or even to assess it, ambiguities are mounting up [8, 9].

An ability to reflect is not only necessary for efficient use of feedback in medical education [10], it is also essential for clinical practice, and it has been argued that the ability to reflect on one’s own role and performance is a key factor in reliable self-assessment and expertise development [11]. It therefore seems logical to teach and assess reflection in specialist training [12, 13].

Such a reflection ability, however, easily becomes an objective beyond the measurable [8, 9].

Traditional assessment methods face problems in assessing the complex clinical competencies that doctors are expected to handle [14, 15]. In such complex competencies the ability to self-assess and reflect is a crucial part but trainee doctors may experience insufficient attempts to measure reflection as counterproductive or even harmful [16]. Since assessment of complex competencies is difficult, authors have suggested to shift focus from traditional summative assessment towards a more formative feedback and how to support learning [15].. Furthermore despite the challenge to assess reflection, it is well established that medical education benefits from training that aims to enhance the reflective capacity of the trainees [12].

A qualified attempt to measure reflection during medical training has been made by the Dutch authors Aukes et al. who developed a tool to measure self-reported level of reflection. They concluded that their tool only measures part of the reflection ability [17]. Other approaches to assessing written reflection have been suggested, e.g. REFLEC [18], and they show positive effects and possibilities in assessing reflection [19]. In continuing medical development collaborative reflection based on verbal exchange of thoughts and experiences has a long and strong tradition [20] and educational beneficial outcomes have been reported [2]. Based on the above-mentioned experiences we therefore assume that reflection ability can be enhanced and assessed by a combination of written reflections and verbal dialogue between trainer and trainee.

Realizing that exact measurement of reflection is impossible, but at same time respecting the importance of the concept, we have tried to develop a new workplace-based procedure, or tool, to enhance and assess reflection through systematic trainer–trainee discussions. We have named this tool “Global Assessment of Reflection ability” (GAR).

In order to validate the tool, we conducted a study in three parts addressing three research questions:

- Is GAR acceptable?
- What is the feasibility and face validity of GAR?
- What is the construct validity of GAR, i.e. does it assess the intended construct of reflection?

## Methods

The development of the assessment tool was based on an understanding of reflection in line with the definition presented by the AMEE guide 44:” Reflection *is a metacognitive process that creates a greater understanding of both the self and the situation so that future actions can be informed by this understanding. Self-regulated and lifelong learning have reflection as an essential aspect, and it is also required to develop both a therapeutic relationship and professional expertise.”* [12].

The tool is primarily focusing on formative assessment for further learning but also provides decision support for a summative yes/no assessment of the ability to reflect in relation to a specific complex competency. This is in line with a modern approach to assessment in medical education in which assessment is focused on the learning of trainees and at the same time used to *“support trainers in taking entrustment decisions by contemplating their “gut-feeling” with information from assessments*” [21].

### Description of GAR

The GAR tool includes two phases.

1. Preparation: The trainee produces a mind map or similar written presentation in a concept formation process addressing a specific, complex competency. The trainee is given 1–2 weeks for the preparation and uses the description of the competency in the curriculum and possible portfolio notes as inspiration.
2. Structured discussion: The trainee gives a brief presentation of his/her mind map/written presentation. This serves as the basis for a structured discussion between trainer and trainee. The discussion includes references to knowledge and experiences that the trainee has obtained in relation to the assessed competency.

During the discussion the trainer assesses the following:

- Does the trainee show ability to reflect on the problem/competency and on his/her own role as a GP?
- Does the trainee demonstrate relevant analytical skills concerning the problem/competency?
- Is the trainee able to participate open minded in a dialogue and demonstrate relevant flexibility?

The focus of the discussion is on formative aspects leading to a plan for further learning, but it also provides decision support for the trainers’ summative pass or fail assessment of a specific competency.

The tool was introduced in the Danish general practice (GP) specialist training in 2014. In several of the complex competencies in the Danish Curriculum the GAR is an integrated part of the global assessment. These are competencies where the ability to reflect is crucial for mastering the competency. An example of a complex competency and a corresponding mind map is showed in appendix 1.

We conducted a study in three parts addressing three research questions:

### Acceptability

The first part of the study addressed the acceptability of the tool. 750 GP trainers from two of Denmark’s five regions (The Region of Southern Denmark and Region Zealand) were introduced to the tool on tutor courses during 2014 and 2015 as part of the nationwide implementation of a new GP curriculum. At the end of each of the in total 32 courses the participants were systematically asked “What do you think of the reflection tool?”. All answers were written down by the teachers, analysed using systematic text analysis, and condensed into main categories of statements [22].

### Feasibility and face validity

The second part of the study addressed feasibility and face validity of the tool.

A questionnaire survey was conducted among GP trainees and GP trainers, who were supposed to have used the new tool in real life because they had had a trainee after the implementation of GAR in the training programme.

Based on the results of the first part of the study we developed a questionnaire containing 12 closed questions regarding demographics, practical conditions, usefulness, and relevance of the tool. One open-ended question collected general views concerning GAR. The questionnaire could be answered within 5 min.

The questionnaire was pilot tested for understandability and content validity in a think-aloud process by three GP trainees and three GP trainers [23]. No significant changes were made after the pilot. The questionnaire can be seen in Appendix 2.

The answers to the open-ended question were condensed and analysed using Systematic Text Condensation [22] and summarized in three categories: Positive comments regarding GAR, negative comments regarding GAR, and comments concerning workload and general reluctance against schedules and mandatory learning and assessment methods.

In 2015 the questionnaire was sent by email to the 354 GP trainers and 216 GP trainees from The Region of Southern Denmark and Region Zealand, who were supposed to have used the tool in real-life clinical setting. Reminders were sent after 2 weeks. 5 GPs and 2 trainees were no longer working as GP trainers or GP trainees and were excluded from the study.

### Construct validity

The third part of the study addressed the construct validity of the tool. We investigated whether relevant reflection was demonstrated by the GP trainees during authentic structured discussions using GAR.

In order to base our analysis and rating on an operational understanding of reflection, we chose to use the SOLO (Structure of Observed Learning Outcomes) taxonomy (Biggs and Collis 1982). This taxonomy operates with five levels of understanding; 1) pre-structural, 2) uni-structural, 3) multi-structural, 4) relational, 5) extended abstract [24]. Level 4 and 5 describe an understanding where different elements are integrated and conceptualized. We defined level 4 and 5 as reflection.

A multi-professional team of six educational experts developed and validated rating schemes applying the SOLO taxonomy onto two of the complex competencies to be assessed by GAR in the Danish GP specialist training programme.

We translated the five levels of the SOLO taxonomy into Danish. Then we split each of the two competencies into five observable objectives. The two rating schemes were constructed combining the descriptions of the SOLO levels 1–5, the five observable objectives, and a global rating for each of the competencies. We scored the highest obtained SOLO level that was reached concerning each objective (Appendix 3). If some of the objectives were not addressed in the discussion no rating would be given.

The two rating schemes were piloted in a process where three experienced researchers each rated two authentic audio recorded structured discussions. The researchers discussed face-, content- and construct validity and found the rating schemes reliable and fit for purpose. An inter-rater variation analysis showed only few and minor differences in rating.

To obtain audio recorded authentic structured discussions for our study we had educational coordinators throughout Denmark repeatedly asking all relevant GP trainers and trainees to send their recorded discussions to the researchers, audiotaped via smartphone and sent by mail. This was done over the course of 1 year. The two rating schemes were used by two researchers to rate the authentic structured discussions. The two researchers rated independently and afterwards negotiated an agreement to reach the final rating of the discussions.

### Statistics

Descriptive statistics and kappa inter-rater variability analysis was calculated in STATA 16.0.

## Results

### Acceptability

In the part of the study addressing acceptability we condensed the answers from the GP trainers into the following statements: “The tool makes good sense”, “The tool seems to be feasible”, “The tool is assumed to be a way to improve quality of trainer-trainee discussions”, and “The tool is a way to obtain an understanding of the trainee’s ability to reflect”. Only one of the 750 trainers expressed negative views finding the instrument to be “waste of time and unnecessary”.

Quotations: “*GAR can make the trainee reflect on own practice”, “The mind-map is useful in structured feedback when it comes to complex competencies”.*

### Feasibility and face validity

In the questionnaire survey addressing feasibility and face validity we received a total of 301 responses, a response rate of 58% (201/349) for GP trainers and 47% (100/214) for GP trainees. The majority of the respondents were female (56% (112/201) of GP trainers and 78% (78/100) of GP trainees). The GP trainers’ average age was 52 years. The trainees’ average age was 31 years. We have no demographic data about non-responders.

88% (264/301) of the trainers and trainees reported to be familiar with GAR, 37% (110/301) had used the tool in vivo. 79% (50/63) of the GP trainers and 72% (34/47) of the GP trainees who had used GAR found it useful or very useful. 81% (51/63) of the GP trainers and 64% (30/47) of the trainees who had used GAR found it relevant or very relevant (Table 1).

**Table 1:** Questionnaire survey

Usefulness and relevance	GP Trainers (63)	GP Trainees (47)
**Very useful**	19% (12/63)	19% (9/47)
**Useful**	60% (38/63)	53% (25/47)
**Less useful**	11% (7/63)	19% (9/47)
**Not Useful**	5% (3/63)	2% (1/47)
**Do not know**	5% (3/63)	6% (3/47)
**Very relevant**	17% (11/63)	11% (5/47)
**Relevant**	63% (40/63)	53% (25/47)
**Less relevant**	10% (6/63)	21% (10/47)
**Not relevant**	3% (2/63)	9% (4/47)
**Do not now**	6% (4/63)	6% (3/47)

Responder’s view of GAR’s usefulness and relevance as at tool for assessing reflection

The majority of the GP trainers (73% (46/63)) used less than 20 min for preparation before the structured discussion. 68% (32/47) of the trainees used less than 30 min for preparation. 74% (81/110) of the structured discussions were completed in 30 min or less.

The open-ended question in the survey was answered by 19% of the respondents (57/301). Of these 42% gave positive statements regarding the tool, 17% gave negative statements regarding the tool and 40% gave general statements in relation to education or other issues.

The condensed positive statements expressed the following opinions: “GAR stimulates reflection and formative assessment that strengthens professional development and in-depth understanding”. “It is relevant for some complex competencies and helps the trainers to generate explicit language about issues that previously have been assessed only by implicit impressions”. “The tool is suitable for strengthening the competent trainee but also suitable to help the trainer when in doubt about the summative assessment i.e. pass-fail decision”.

The condensed negative statements expressed the following opinions: “The tool aims at measuring the unmeasurable and is trying to plan things that can’t be planned”. “It is time consuming and a waste of time”. “Unstructured assessment without mandatory tools is preferred”.

The condensed general statements expressed the following opinions: “Mandatory use minimizes motivation for using new learning or assessment methods”. “New demands concerning education combined with high work-load in general practice gives less room for implementing new methods”. Requests for less control in education were stated from both trainers and trainees.

### Construct validity

Eight authentic structured discussions were rated according to the developed rating schemes. Kappa inter-rater agreement analysis showed 83% agreement (Kappa 0.70, SE 0,14, *p* < 0.001). This indicates a high degree of agreement. The two researchers negotiated and reached an agreement of the final rating (Table 2).

**Table 2:** Audio ratings

**Recording**	**Item 1** **Patient** **Encounter**^**1**^	**Item 2** **Patient** **encounter**^**1**^	**Item 3** **Patient encounter**^**1**^	**Item 4** **Patient** **encounter**^**1**^	**Item 5** **Patient encounter**^**1**^	**Global rating**
Trainee1	5	5	4	n/a	5	**5**
Trainee 2	4	n/a	3	n/a	4	**4**
	**Item 1** **Educator**^**2**^	**Item 2** **Educator**^**2**^	**Item 3** **Educator**^**2**^	**Item 4** **Educator**^**2**^	**Item 5** **Educator**^**2**^	**Global rating**
Trainee 3	4	5	5	n/a	5	**5**
Trainee 4	4	5	5	5	5	**5**
Trainee 5	5	5	4	5	5	**5**
Trainee 6	4	5	5	5	5	**5**
Trainee 7	4	3	3	3	4	**3**
Trainee 8	n/a	n/a	5	5	5	**5**
**Mean**						**4.6**

Rating of the 8 audio recordings of real-life assessments according to the SOLO taxonomy

1 = pre-structural, 2 = uni-structural, 3 = multi-structural, 4 = relational, 5 = extended abstract (ref). Level 4 and 5 defined as reflection

n/a = not rated

^**1)**^ Trainee 1–2. Competency: “Different types of patient encounters” which includes the ability to use the different kinds of patient encounters (consultation, home visit, telephone call etc.) adequately and embrace individual, social, cultural and contextual considerations

^**2)**^ Trainee 3–8. Competency: “Educator” including skills for teaching colleagues and staff considering aspects of target group, prior qualifications, and factors enhancing or inhibiting learning

The mean global rating was 4,6 on the 5-point scale based on the SOLO taxonomy, meaning that the 8 structured discussions on average ranked between what is called “relational” and “extended abstract” in the SOLO taxonomy which resembles our defined level of reflection.

## Discussion

### Principal findings

Our study shows an initial high acceptance to the introduction of GAR among Danish GP trainers. However, the responses in the following survey were more diverse.

The feasibility and face validity of GAR seems high among the trainees and trainers who have used the tool. Both GP trainers and trainees found the tool useful and relevant. The trainers and trainees reported that the tool stimulates reflection in relation to complex competencies and helps trainers assess complex competencies by generating explicit language about matters previously only informed by implicit impressions. Compared to the prior situation with only implicit and intuitive judgment the tool seems to be suitable for strengthening the competent trainee. However, the implementation of the tool as part of the daily work-based assessment is proceeding at a relatively slow pace. We also found some general resistance against structured educational initiatives among both trainers and trainees and GAR was met with skepticism by some, because of time constraints in busy clinical settings and a reluctance due to an impression of rising demands of control in the society.

Ratings of the audio recordings showed an acceptable inter-rater variability and it demonstrated reflection in the trainer-trainee discussions concerning complex competencies.

We conclude that GAR has a sufficient degree of construct validity, i.e. that appropriate assessment of the trainee’s ability to reflect can be made using the tool.

Our tool was seen by most trainers and trainees as acceptable, feasible, and having face and construct validity - characteristics that are essential for success in assessment in medical education [25]. In another Danish study GAR was found to be less used than the other assessment methods in the specialist training programme, but was found similarly valuable by those who used it [26].

Some trainers and trainees were skeptical towards GAR. We know from other studies that some resistance can be expected from experienced clinicians presented with attempts to map uncertainty, or potential educationally reductive approaches to complex competencies [2, 14].

We find this a relevant reservation, which should be considered when improving our reflection tool. Nevertheless the relevance of attempts to enhance and assess the ability to reflect is well supported by literature [12].

Attempts to support reflective thinking in specialist training is not new. Written clinical incidences via an online portfolio have been used in Denmark since 2004 and has been proven beneficial for some but not all trainees [27]. In our study, however, we have focused on assessing verbal trainer-trainee discussions with a formative focus based on a prior mind-mapping and concept formation process where the trainee creates a written presentation. The literature supports the use of mind-maps and trainer-trainee discussions to enhance reflection [28]. Reflection-driven development is also seen in verbally founded reflective learning groups [2, 20]. In expertise development theories the ability to reflect is prerequisite for competence development and support our educational focus on reflection [29, 30].

We think these findings support our attempt to enhance and assess reflection in medical education.

### Strengths and weaknesses

In the acceptability part of the study we invited 760 of ordinary trainers to test the tool in vitro at our train-the-trainer courses. The trainers came from two different parts of the country having participated in different trainer courses with different instructors.

In spite of time constraints and the reported in-born skepticism towards new assessment methods, the vast majority found the tool acceptable. However, the result was based on initial experiences obtained in a training session and answers given verbally, which might influence the response.

To investigate feasibility and face validity, we asked ordinary trainers and trainees via a questionnaire survey to evaluate the tool after having tried it in their own practices in authentic settings. We invited both GP trainers and trainees to participate in the survey to engage both perspectives and we invited participants from two different educational regions to avoid bias according to personal or regional issues. The response rate was acceptable both for GP trainers and trainees.

In the survey based on GP trainers and trainees after a single non-guided first-time use, we would expect difficulties and resistance, but we found a high degree of perceived usefulness and relevance among the users of the tool. However, a substantial number of participants had not yet used the tool at the time of the survey which enhances the risk of selection bias.

Investigating construct validity, one would often test against a golden standard. We did not have a golden standard to test our tool against, though, and we had to find another approach. We found that the SOLO taxonomy could help us rate reflection as intended. Unfortunately, we have not found literature using the same method in assessing reflection in a clinical setting to support our findings. However, earlier researchers have shown other possible approaches to assess written reflections supporting learning, indicating that clinical reflection can be assessed [17, 19, 27].

We hypothesized that reflection can be graduated into levels from superficial to deep critical reflection and chose the recognized SOLO taxonomy to rate the level of reflection in the trainer-trainee discussions. The rating scheme was thoroughly elaborated by six professionals with different educational background, four physicians, an educationalist, and a psychologist.

We obtained an acceptable degree of inter-rater agreement using the rating schemes. However, we experienced practical difficulties collecting recorded, authentic structured discussions. We had to repeat the invitation to record just to reach the number of 8 recordings, which is quite a small amount of material. We assume the technical challenge in busy daily GP practice combined with some professional shyness account for some of the recruitment difficulties. Therefore, there is undoubtedly a degree of selection bias in our study. But even with our limited material the tool demonstrated measurable reflection in relation to complex competencies.

### Implications and further research

We assume that the GAR tool could be relevant to apply in other settings than Danish GP training, but larger scale studies are needed to detect level of obtained reflection and the tool’s ability to discriminate among high and low performers in different professional socio-cultural settings. It also needs to be explored whether a tool such as GAR will stimulate more demonstrated reflection than an unstructured but engaged trainer–trainee discussion.

## Conclusions

We have developed an assessment tool (GAR) to enhance and assess reflection. GAR was found to be acceptable, feasible, and relevant by most trainers and trainees. The study indicated that both face- and construct validity is good. GAR seems to be able to enhance the trainees’ ability to reflect and provide a good basis for assessment in relation to complex competencies.

## Figures and Tables

**Fig. 1:**
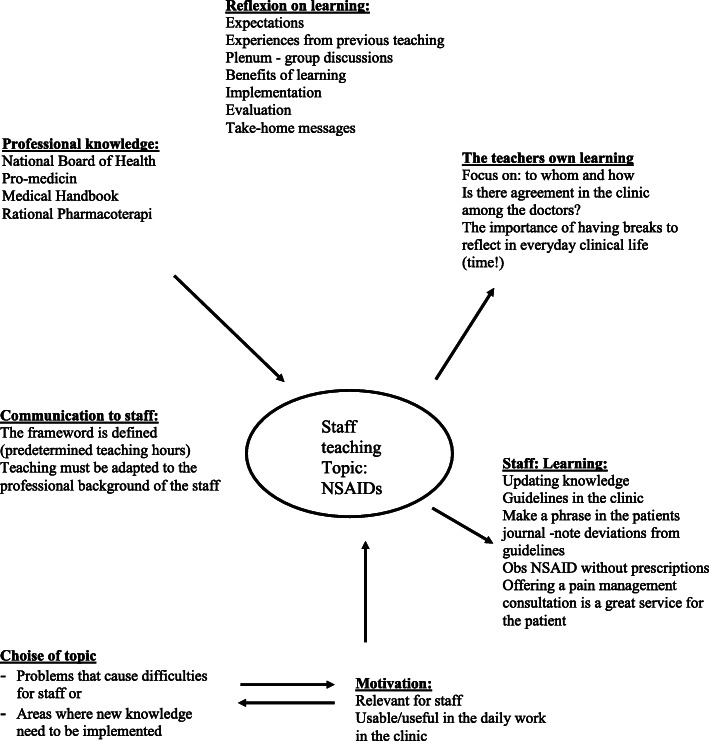
Example of a corresponding mind map. Mind-map published with accept from the participants

## Data Availability

The datasets used and/or analysed during the current study are available from the corresponding author on reasonable request. However, the recorded trainer/trainee conversations are confidential and are therefore not available.

## Appendix 1

**Table 3:** Competency # 8: Teaching. From the Danish curriculum for GP specialist training. Translated from Danish by the authors

Description	Learning strategy	Assessment
The doctor must be able to: Teach colleagues, staff, and non-health educated persons. Communicate professional topics while taking the recipient’s background into account. In a constructive way apply knowledge of factors that can promote learning in the clinic and demonstrate awareness of factors that may inhibit learning. Demonstrate awareness of the fact that the patient - when taking part in teaching - can be both the teacher and the learner. Role: Scholar	Participation in internal teaching in clinic. Participation in specialty training courses.	Assessment by Global assessment of reflection ability (GAR)

## Appendix 2 Electronic questionnaire

**1. What is your position?**

Trainer

Trainee in introductory year

Trainee in specialist training

Other (please specify)

**2. What is your gender?**

Woman

Man

**3. What is your age?**

<25 years

25 - 35 years

36 - 45 years

46 - 55 years

56-65 years

> 65 years

**4. Do you know the Global Assessment of Reflection ability tool (GAR)?**

Yes, and I have used GAR

Yes, but I have not yet used GAR

No, I do not know GAR

Other (please specify)

**5. How did you and your trainer/trainee prepare before using GAR?**

The trainer prepared in advance

The trainee prepared in advance

Both the trainer and trainee prepared in advance

We used GAR without preparation

We have not used GAR

**6. How long time have you spent preparing for GAR? (If you have used GAR more than once please indicate the average time for preparation)**

Less than 10 minutes

10 - 20 minutes

21 - 30 minutes

31 - 40 minutes

41 - 50 minutes

51 - 60 minutes

More than 60 minutes 25

I have not prepared for GAR

Other (please specify)

**7. How long time have you spent on the structured discussion as part of GAR with your trainer/trainee? (If you have used GAR more than once please indicate the average time)**

Under 10 minutes

10 - 20 minutes

21 - 30 minutes

31 - 40 minutes

41 - 50 minutes

51 - 60 minutes

More than 60 minutes

We have not had a GAR structured discussion, please state reason:

**8. How do you rate the usefulness of GAR as a tool for assessing the ability of a trainee to reflect?**

Very useful

Useful

Not so useful

Not useful at all

Do not know

Have not used GAR

**9. ONLY FOR TRAINEES: Have you used the guiding questions and mind map examples for trainees regarding GAR? (see the guiding questions and mind maps examples via the link below) http://www.dsam.dk/flx/courses/student/competencevaluation/guide_conferences_of_requirement/)**

Yes, and the questions/mind map examples were helpful

Yes, but the questions and mind map examples were not as helpful as I had hoped for

No, I did not need the questions or mind map examples

No, I was not aware of the questions and mind map examples

Other (please specify)

**10. ONLY FOR TRAINERS: Have you used the assessment criteria for trainers regarding GAR? (See the assessment criteria via the link below) http://www.dsam.dk/flx/uddannelse/fremuddannelse_i_almen_medicin/kompetencevurdering/vejlederssamtale_vurdering_af_refleksionsevne/)**

Yes, and the assessment criteria were helpful

Yes, but the assessment criteria were not as helpful as I had hoped for

No, I did not need the assessment criteria

No, I was not aware of the assessment criteria 26

Other (please specify)

**11. How do you rate the relevance of GAR as a method for assessing reflection?**

Very relevant

Relevant

Not so relevant

Not relevant at all

Do not know

Have not used GAR

**12. If you have any comments on GAR, suggestions for application, experience with the application or anything else you are welcome to write this here:**

## Appendix 3

**Table 4:** Rating scheme for structured discussions

	QUANTITATIVE		QUALITATIVE	
	SOLO 2	SOLO 3	SOLO 4	SOLO 5
**Ability to reflect on topical issue and one’s own work, role and options.**	”Uni-structural”	”Multi-structural”	”Relational”	”Extended abstract”
**Educator**	Memorise, identify, recognise, count, define, find label, match, name, quote, recall recite, order, tell, write imitate	Classify, describe, list, report, discuss, illustrate, select, narrate, compute, sequence, outline, separate	Apply, integrate analyse, explain, predict, conclude, summarise, review, argue, transfer, make a plan, characterise, compare, contrast, differentiate, organise, debate, make a case, construct, review and rewrite, examine, translate, paraphrase, solve a problem	Theorise, hypothesise, generalise, reflect, generate, create, compose, invent, originate, prove from first principles, make an original case, solve from first principles
Considerations about prior qualifications, level, and needs in the target group				
Considerations about adaptation to prior qualifications, level, and needs in the target group				
Considerations about teaching method(s)				
Considerations about factors enhancing and inhibiting learning				
Considerations about own work, role and options in relation to teaching colleagues and staff				
Global rating				
**Different types of patient encounters**	Memorise, identify, recognise, count, define, find label, match, name, quote, recall recite, order, tell, write imitate	Classify, describe, list, report, discuss, illustrate, select, narrate, compute, sequence, outline, separate	Apply, integrate analyse, explain, predict, conclude, summarise, review, argue, transfer, make a plan, characterise, compare, contrast, differentiate, organise, debate, make a case, construct, review and rewrite, examine, translate, paraphrase, solve a problem	Theorise, hypothesise, generalise, reflect, generate, create, compose, invent, originate, prove from first principles, make an original case, solve from first principles
Considerations about implications for patient communication in different kinds of patient encounters (consultation, phone calls, e-mail, home visits)				
Considerations about own role in relation to home visits				
Considerations about impact of individual, societal, cultural, and contextual circumstances in relation to patient communication and presentation of symptoms				
Considerations about patient communication using an interpreter				
Considerations about impact on communication in case of interruptions, need for prioritising several simultaneous tasks, communication about several topics at a time, and communication with numerous persons at a time				
Global rating				

## Abbreviations

GAR: Global assessment of reflection
GP: General practice
